# CHARMM-GUI Hybrid ML/MM Builder for Hybrid Machine Learning and Molecular Mechanical Modeling and Simulations

**DOI:** 10.1021/acs.jcim.6c00060

**Published:** 2026-03-09

**Authors:** Florence Szczepaniak, Donghyuk Suh, Wonpil Im

**Affiliations:** Department of Biological Sciences, 1687 Lehigh University, Bethlehem, Pennsylvania 18015, United States

## Abstract

Recent advances in machine learning (ML) have enabled new developments in molecular dynamics simulation. Neural network potentials (NNPs) trained on quantum mechanical (QM) data provide highly accurate descriptions of drug-like molecules. Analogous to a QM and molecular mechanical (QM/MM) approach, hybrid ML/MM simulations employ NNPs to describe a localized region of the system, such as a ligand, while the rest of the system is treated using classical MM force fields. This hybrid framework enables simulations of protein–ligand complexes with near-QM accuracy for the ligand at a substantially reduced computational cost. CHARMM-GUI *Hybrid ML/MM Builder* automates the preparation of system and input files required for hybrid ML/MM modeling and simulation. This new module generates all necessary files to simulate protein–ligand complexes in solution or membrane using TorchANI-AMBER and OpenMM-ML. Currently supported NNPs include MACE and ANI. In this paper, we present *Hybrid ML/MM Builder* and representative application systems that demonstrate its usage and capabilities.

## Graphical abstract

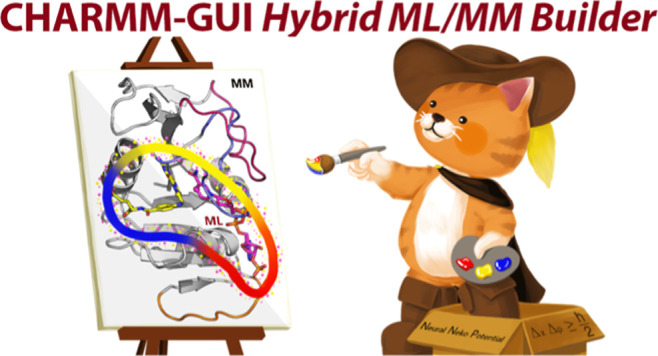

## Introduction

Molecular mechanical (MM) simulation requires a force field to describe intra- and intermolecular interactions within a system.^1^ These force fields have been developed empirically, and the description of large biomolecules such as proteins,^2,3^ nucleic acids,^4^ carbohydrates,^5^ and lipids^6,7^ has reached a level of accuracy that enables the investigation of a wide range of biomolecular phenomena.^8,9^ Smaller, drug-like molecules are typically parametrized using general force fields such as CGenFF,^10^ GAFF,^11,12^ or OPLS.^13,14^ Despite substantial progress in the force field development, systematic errors persist, particularly in the calculations of thermodynamic quantities.^15^ Accurate ligand descriptions are therefore essential for reliable binding free energy calculations in drug discovery applications, so making the continued improvement of ligand parameters a topic of major interest.^16^

Several approaches have been developed to improve ligand descriptions. For example, the Drude polarizable force field offers a more accurate description of molecular polarizability.^17^ An alternative strategy is the use of QM (quantum mechanical)/MM simulations, in which most of the system is treated with classical force field parameters, while a selected region, typically the ligand, is described using QM.^18^ This approach enables a more accurate representation of ligand energetics and interactions with the surrounding environment.^19–21^ However, a high computational cost of QM/MM simulations severely limits the simulation length.

More recent developments leverage artificial intelligence (AI) to learn from QM calculations and construct improved force fields.^22^ Large data sets such as QM7b^23^ or SPICE^24^ provide millions of molecular conformations evaluated at a QM level. Neural network potentials (NNPs) including MACE^25,26^ or ANI^27,28^ are trained on these data sets to accurately describe the energetics of drug-like molecules. This progress has enabled hybrid ML/MM simulations, in which the ligand is described by NNP, while the remainder of the system is treated using a classical force field.^29–31^ Such hybrid approaches offer near-QM accuracy for the ligand at a fraction of the computational cost of traditional QM/MM simulations.

CHARMM-GUI^32^ is a web-based platform designed to generate system and input files for a wide range of biomolecular simulations. In this paper, we present a new CHARMM-GUI module, *Hybrid ML/MM Builder*. Without an integrated workflow, hybrid ML/MM simulation setup commonly involves manual coordinate extraction for the ML region, preparation and verification of topology/parameter files for the surrounding MM system, handling of ML/MM interface definitions, and generation of engine-specific control/input files. The CHARMM-GUI *Hybrid ML/MM* Builder automates these tasks within a standardized web-based pipeline, reducing manual intervention and helping users avoid common setup errors. The current implementation supports OpenMM^33^ and AMBER^34–36^ as simulation engines. CHARMM-GUI provides ready-to-use input files employing NNPs from the MACE^25,26^ and ANI^27,28^ families, and also allows users to modify the generated files to incorporate custom NNPs. A tutorial video demonstrating its use for a protein–ligand complex in solution is available on the CHARMM-GUI Web site (https://www.charmm-gui.org/demo/mlmm). To illustrate the applicability and capabilities of *hybrid ML/MM Builder*, we present three representative systems: the catalytic domain of human phosphodiesterase in complex with tadalafil (PDB ID 1XOZ),^37^ the inactive epidermal growth factor receptor (EGFR) tyrosine kinase domain bound to erlotinib (PDB ID 4HJO),^38^ and a G-protein-coupled receptor structure bound to an agonist (PDB ID 6KPC).^39^

## Methods

### Hybrid ML/MM Simulations

In hybrid ML/MM simulations, the total potential energy of a system is expressed as

$$V\left(r\right)=V_{MM}\left(r_{MM}\right)+V_{ML}\left(r_{ML}\right)+V_{MM/ML}\left(r_{MM/ML}\right)$$

where V(r) denotes the potential energy of the whole system, $V_{MM}\left(r_{MM}\right)$ corresponds to a classical MM potential acting on the MM region, $V_{ML}\left(r_{ML}\right)$ represents an NNP describing the ML region, and $V_{MM/ML}\left(r_{MM/ML}\right)$ accounts for nonbonded interactions between the MM and the ML regions.^30,31^ The form of the coupling term $V_{MM/ML}$ depends on the chosen embedding scheme for the ML region. The most straightforward embedding approach is mechanical embedding, where the electrostatic interactions between the ML and MM regions are evaluated using fixed partial charges.^29,40^ More advanced embedding NNP schemes that incorporate polarization effects and charge redistribution are under active development.^41,42^ At present, *Hybrid ML*/*MM Builder* generates system and input files based on mechanical embedding when resorting to OpenMM. Using TorchANI-Amber, the *Hybrid ML/MM Builder* generates input files based on mechanical embedding if the chosen NNPs are ANI-1ccx or ANI-2*x*, but can also support simulations with variable charges with the NNP ANI-MBIS.^43^ In addition, the definition of the ML region is constrained by the capabilities of the selected NNP. Both MACE and ANI impose restrictions on supported atom types, with current ANI models limited to C, N, H, O, and selected halogens, as well as constraints on the total molecular charge.^26,27^ Furthermore, these NNPs do not support covalent coupling between ML and MM regions. Consequently, *Hybrid ML*/*MM Builder* does not currently allow the ML region to be defined as a fragment of a covalently connected molecule or across regions involving changes in bond order.

### Workflow of *Hybrid ML*/*MM Builder*

The overall workflow of *Hybrid ML*/*MM Builder* is shown in Figure 1. The workflow is analogous to that of CHARMM-GUI *QM*/*MM Interfacer*.^44^ The entry page of the module provides direct links to OpenMM-ML^33^ and TorchANI-Amber^43,45,46^ repositories. The workflow begins with *PDB Reader and Manipulator,* ^32,47,48^ where users may upload a structure or retrieve one from RCSB Protein Data Bank. At this stage, users specify whether the system will be simulated in solution or embedded in a membrane, select molecular components such as proteins and ligands, and apply various modifications such as modeling missing residues, mutation, protonation, glycosylation, and other post-translational modifications as needed. The second step involves system assembly using either Solution Builder^49^ or Membrane Builder,^50,51^ depending on the chosen environment. In this step, users define solvent conditions or membrane composition, including lipid types and bulk components, using the standard CHARMM-GUI protocols.

**Figure 1:**
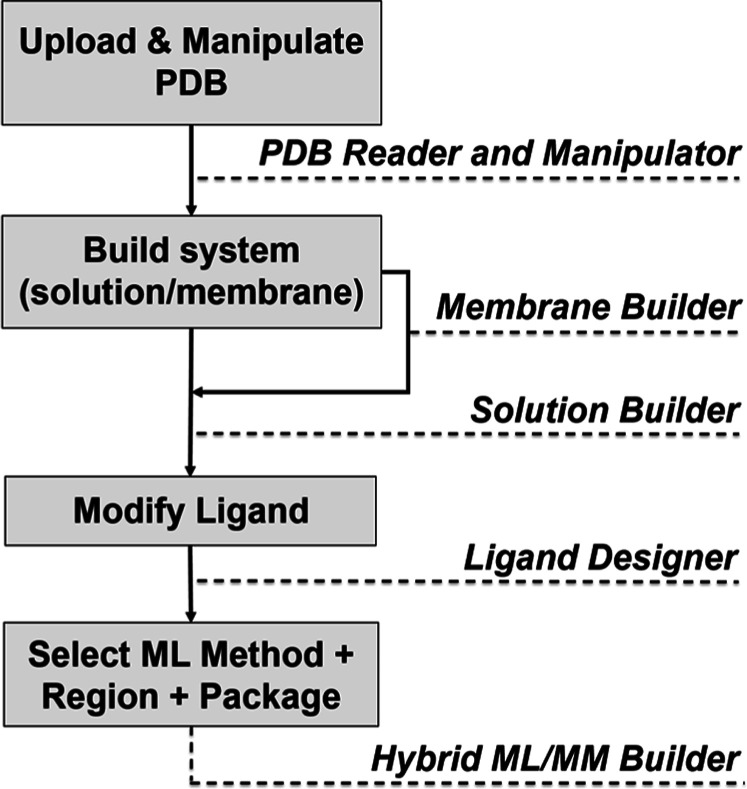
Schematic workflow of *Hybrid ML*/*MM Builder*.

In the third step, ligand preparation is performed using functionality shared with Ligand Designer^52^ (Figure 2A). Ligands may be inspected and modified using the NGL viewer^53^ and the Marvin JS interface, respectively. During this step, users can manually modify the atoms or the bond order of ligand. Since NNP applicability is limited by supported atom types and charge states, users are responsible for ensuring that the modified ligand is compatible with the selected NNP requirement with MACE or ANI. A warning appears if the ligand is not compatible with the NNP, but users can bypass it if they provide their own NNP. In the final page, users select the force field for the MM region, the NNP for the ML region, and the simulation engine (Figure 2B). The visualization of the ML region is done by a magenta colorization of the ligand.

**Figure 2:**
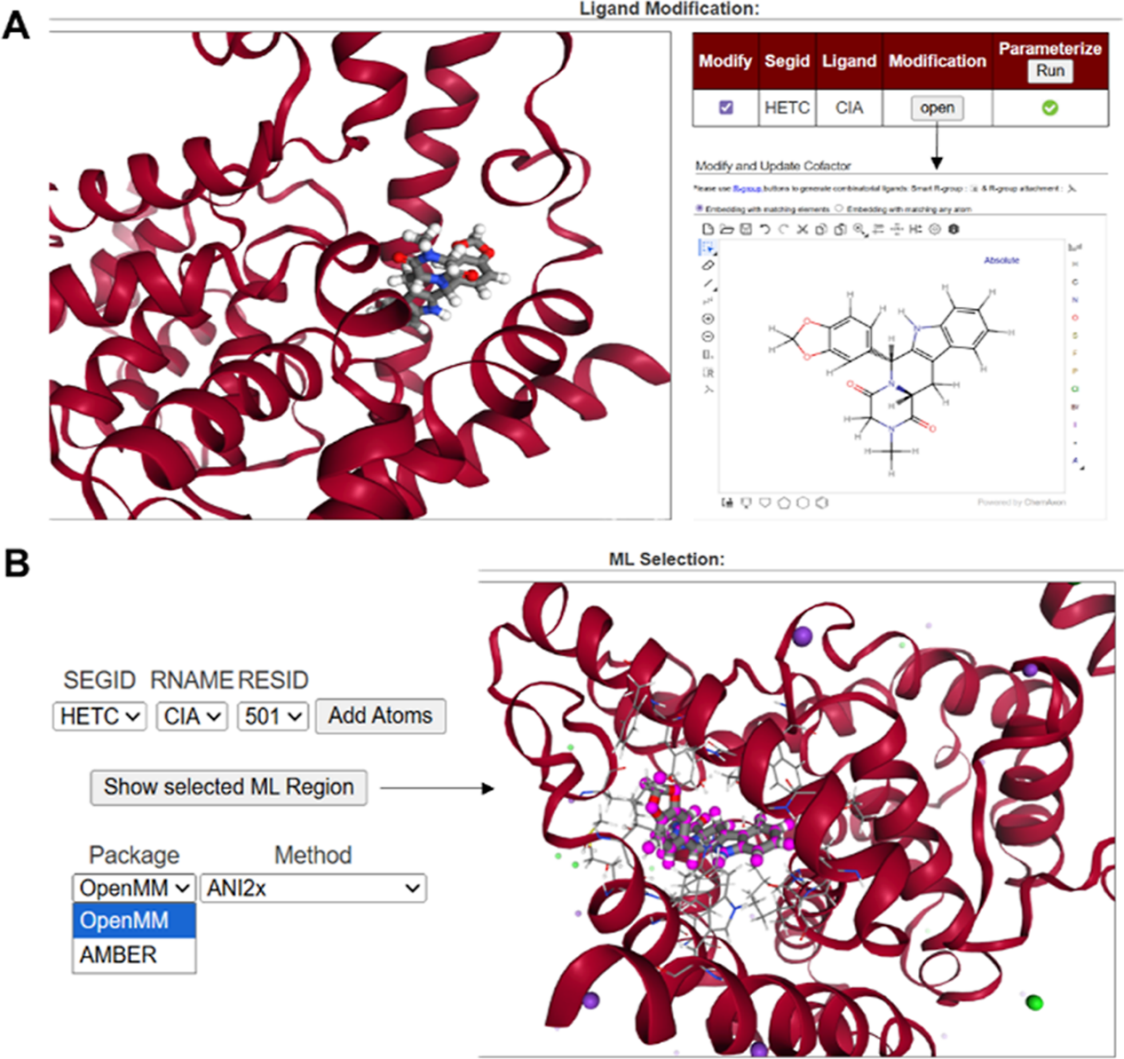
(A) Ligand selection and modification of the ligand CIA in PDB ID 1XOZ. (B) ML region, package, and method selection followed by visualization of the selected ML region.

Table 1 summarizes the currently supported simulation packages and NNPs. The classical force fields available for the MM region are currently limited to the AMBER force fields.3 ^54,55^ Since the ligand is treated using an NNP, its parameters cannot be modified. While *Hybrid ML*/*MM Builder* currently provides built-in support for MACE and ANI potentials, users may adapt the generated input files to employ alternative NNPs, provided that compatible models are supplied.

**Table 1:** List of Packages and NNPs Supported by *Hybrid ML*/*MM Builder*

packages	neural network potentials
openMM^33^	ANI-1ccX, ANI-2*x*,^26,56,28^ MACE^26^
torchANI-Amber^34–36,43,45,46^	ANI-1ccX, ANI-2*x*, ANI-MBIS^27,28,56^

### Computational Details

Three representative systems were selected to demonstrate the functionality and applicability of *Hybrid ML*/*MM Builder*. The first system is the catalytic domain of human phosphodiesterase in complex with tadalafil (PDB ID 1XOZ),^37^ a protein–ligand complex in aqueous solution. The tadalafil ligand is compatible with both MACE and all currently supported ANI models. The complete workflow for generating input files for this system is demonstrated in an instructional video available on the CHARMM-GUI Web site. The second system is inactive EGFR tyrosine kinase domain bound to erlotinib (PDB ID 4HJO),^38^ which is also simulated in solution and features a ligand compatible with all supported NNPs. This system has previously shown to exhibit difference in ligand behavior when parametrized using CGenFF versus ANI,^31^ making it a suitable example to highlight the advantages of ML-based ligand descriptions. The third system is an agonist bound GPCR (PDB ID 6KPC)^39^ that serves to demonstrate the capability of the module to handle membrane protein systems. For this case, simulations were performed for both the full protein–ligand complex in a bilayer and the ligand in solution.

All systems were built with the settings and parameters provided by *Hybrid ML*/*MM Builder.* The classical MM region was described using the AMBER force field available on the .xml format to make the compatibility with OpenMM easier.^33^ The protein was parametrized with the ff19SB^3^ force field, the lipids were parametrized with Lipid17, and OPC^57^ water model was used. For comparison, additional simulations were performed in which the entire system was treated using the classical MM force field. In these simulations, the ligands were parametrized using GAFF. Furthermore, all three ligands were also simulated in aqueous solution in the absence of the protein using both hybrid ML/MM and fully classical MM approaches. These simulations were designed to assess differences in conformational behavior in a less sterically constrained environment when parametrized with GAFF versus ANI-2*x*. The input files for these simulations were generated using *Hybrid ML*/*MM Builder* and Solution Builder with only the ligand selected during the initial stage.

System equilibration was performed using classical MM for both OpenMM and AMBER, followed by production simulations employing the hybrid ML/MM scheme. Production simulations were carried out using OpenMM with the ANI-2*x* with a time step of 1 fs, a temperature of 303.5 K, and a pressure of 1 atm. The 1XOZ system contains 29,500 water molecules, the 4HJO system 32,600 water molecules, and the 6KPC system 60,000 water molecules and a lipid bilayer composed of POPC and POPG in a 3:1 ratio. All systems were neutralized and ionized to a KCl concentration of 150 mM. On one NVIDIA Tesla T4 GPU, using a time step of 1 fs, OpenMM achieves a production rate of 10 ns/day for hybrid ML/MM simulations, enabling routine access to long time-scale sampling. For comparison, the OpenMM MM-only simulation with a 2 fs time step produces more than 80 ns/day. In contrast, TorchANI-AMBER currently operates with sander only for the hybrid ML/MM and produces trajectories at a slower rate (less than 1 ns/day for ML/MM as well as MM using sander).^43^ Structural stability and conformational changes were evaluated by comparing the root-mean-square deviation (rmsd) of the molecules between hybrid ML/MM and classical MM trajectories. Molecular visualization and rmsd calculations were performed using VMD.^58^

## Results and Discussion

### Catalytic Domain of Human Phosphodiesterase in Complex with Tadalafil (PDB ID 1XOZ)

Figure 3A shows the structure of the PDB ID 1XOZ protein–ligand complex where the ligand (CIA) is deeply buried within the binding pocket and is therefore highly conformationally restricted. When simulated in aqueous solution, the CIA ligand (Figure 3B) exhibits substantially larger conformational variability compared with the protein-bound state. Figure S1 shows the rmsd timeseries of the ligand in solution, in complex with 1XOZ and of the protein. Three replicas per systems are shown. The mean value of the rmsd for each system is summarized in Figure S1G. Consistent with the use of the same classical force field for the protein in both simulations, the protein backbone rmsd exhibits comparable behavior in both hybrid ML/MM and fully classical MM simulations. Accordingly, the binding pocket geometry remains stable in both simulation protocols, resulting in similar rmsd profiles for the bound ligand. Notably, the ligand parametrized using GAFF displays greater rmsd fluctuations than the ligand described by ANI-2*x*. Structural inspection reveals that the dominant contribution to this variability originates from changes in the relative orientation of conjugated ring systems.

**Figure 3:**
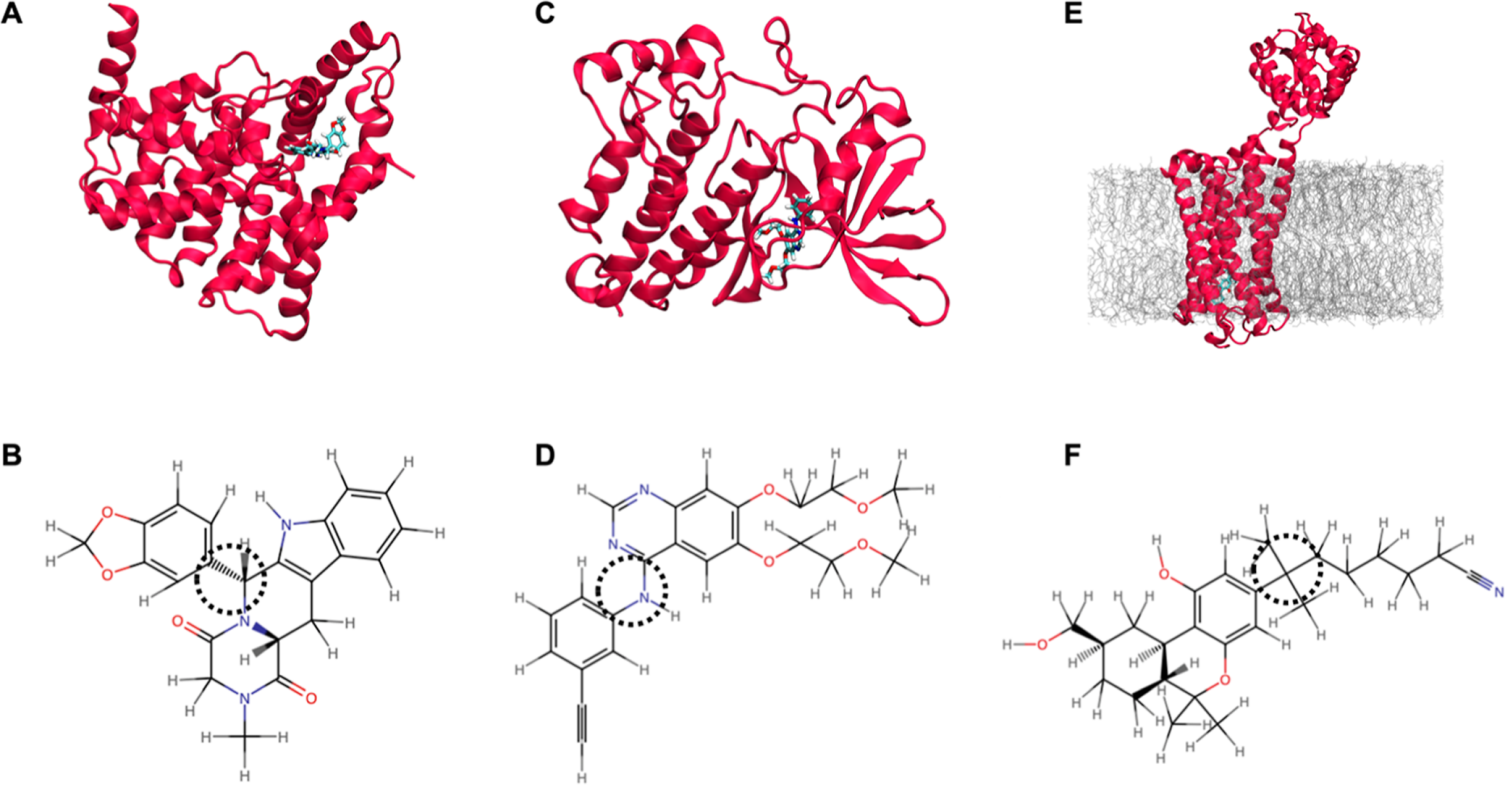
Structures of PDB ID 1XOZ (A), 4HJO (C) and 6KPC (E). The proteins are shown in red, the ligands described using ANI are in cyan and the membrane is in gray. For clarity, water and ions are not shown. Chemical structures of the ligand CIA (B), AQ4 (D) and E3R (F). The dashed circle highlights the torsional region contributing most strongly to rmsd differences.

### The Inactive EGFR Tyrosine Kinase Domain with Erlotinib (PDB ID 4HJO)

For the PDB ID 4HJO system (Figure 3C), the protein rmsd remains similar between the hybrid ML/MM and classical MM simulations (Figure S2), as expected given the identical force field treatment of the protein. The bound erlotinib ligand (AQ4) remains conformationally restricted within the binding pocket but exhibits greater flexibility than the CIA ligand in the PDB ID 1XOZ system. In solution, AQ4 (Figure 3D) displays increased flexibility relative to its bound state, with the GAFF-parametrized ligand showing significantly larger rmsd fluctuations than the ANI-2*x*. This behavior is consistent with previous studies on this ligand using CGenFF and ANI-1ccx with NAMD.^31^ The elevated ligand rmsd fluctuation observed in the classical MM simulation underscores known limitations of the classical force fields in capturing parameters of conjugated groups for flexible molecules.^31,59^

### Agonist Bound GPCR (PDB ID 6KPC)

Protein rmsd values in this GPCR-bilayer system (Figure 3E) is higher in classical MM simulations compared to the hybrid ML/MM simulations, mostly due to the stochastic flexibility in disordered loop regions (Figure S3). The use of NNP yields slightly increased flexibility of the ligand. Since the binding site is deeply buried within the membrane-embedded receptor, however, steric constraints limit the extent of conformational variation, resulting in relatively low rmsd values overall. The E3R ligand (Figure 3F) displays substantially greater flexibility in aqueous solution than in bound state. The ligand described by ANI-2*x* exhibits broader conformational sampling compared to GAFF-parametrized counterpart. The different behavior of the ligand parametrized by ANI-2*x* and by GAFF shows how the NNPs allow a different sampling for the dynamic of the torsion of dihedrals in ligands.^42,59^

Collectively, these results demonstrate that *Hybrid ML*/*MM Builder* can robustly support simulations of diverse protein–ligand systems in both solution and membrane environments. Since ligand behaviors, particularly in solution, exhibit clear and systematic differences between classical and ML–based descriptions, these findings highlight the potential advantages of incorporating NNPs into protein–ligand simulations, especially for organic moieties that are challenging to parametrize accurately using conventional force fields.

## Conclusions

In this work, we introduce *Hybrid ML*/*MM Builder*, a new module in CHARMM-GUI that automates the generation of system and input files for hybrid ML/MM simulations. *Hybrid ML*/*MM Builder* enables users to prepare protein–ligand systems in solution or membrane and to generate ready-to-run simulations using OpenMM and AMBER. The interface facilitates system assembly, selection of simulation engines, and assignment of NNPs for the ML region. The representative systems present the ability of the module to support a range of protein–ligand complexes and produce result consistent with previously published hybrid ML/MM studies. At present, the module supports production simulations of protein–ligand complexes using ANI and MACE NNPs. Extensions to applications such as free energy calculations, treatment of covalently coupled ML/MM regions, and simulations involving charged or long-range electrostatic effects will require the development and broader adoption of next-generation NNPs.^60–63^ Recent efforts leveraging large-scale QM data sets, such as SPICE, aim to address these limitations,^24^ although such models are not yet as mature or widely deployed as ANI or MACE. Further development of *Hybrid ML*/*MM Builder* will focus on adopting the next generation NNPs as well as expanding support to additional simulation engines including NAMD^31^ and GROMACS.^64^

## Supplementary Material



## Data Availability

The data underlying this study, including the input files for model system generation and the corresponding output files, can be reproduced and are freely available in CHARMM-GUI *Hybrid ML*/*MM Builder* (https://charmm-gui.org/input//mlmm).

## Notes

Additional results details (Figures S1–S3), including the rmsd timeseries and mean rmsd values of each series for each simulation system (PDF)
