# Supplementary material for: CHARMM-GUI Hybrid ML/MM Builder for Hybrid Machine Learning and Molecular Mechanical Modeling and Simulations
Source: J Chem Inf Model. 2026 Mar 9;66(6):2960–6. doi: 10.1021/acs.jcim.6c00060 (PMC13014446; doi:10.1021/acs.jcim.6c00060)
Supplement: Supplementary file 1 [file ci6c00060_si_001.pdf]

Supporting information

CHARMM-GUI *Hybrid ML/MM Builder* for Hybrid Machine Learning and Molecular Mechanical Modeling and Simulations

Florence Szczepaniak, Donghyuk Suh, and Wonpil Im*

Department of Biological Sciences, Lehigh University, Bethlehem, Pennsylvania 18015, USA

Corresponding Author Email: Wonpil Im (wonpil@lehigh.edu)

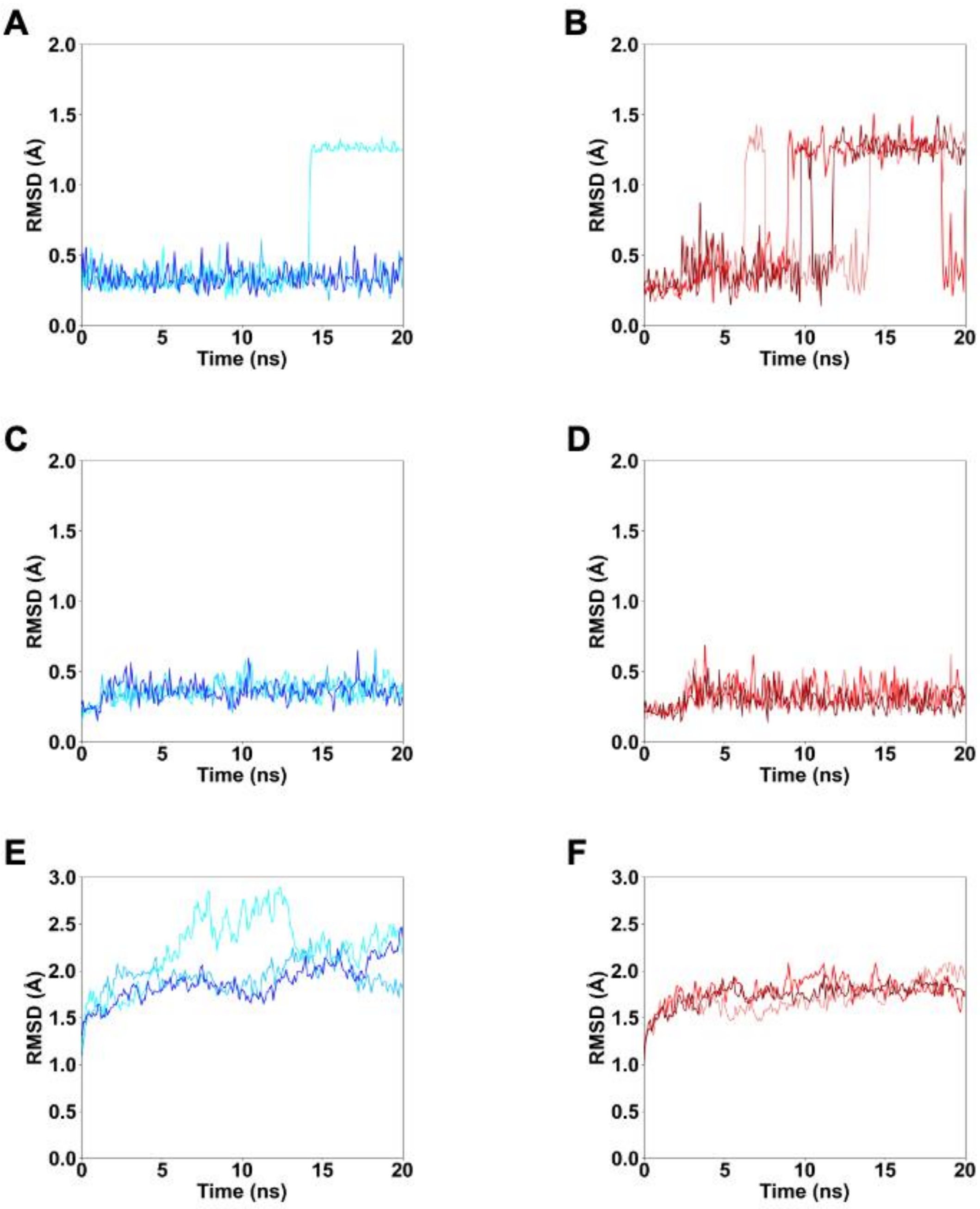

G

System		ML/MM	MM
	CIA in solution	0.44 ± 0.01	0.75 ± 0.3
1XOZ	CIA in complex	0.36 ± 0.03	0.31 ± 0.03
	Protein	1.93 ± 0.03	1.68 ± 0.03

Figure S1. RMSD timeseries of the CIA ligand in solution during the hybrid ML/MM **(A)** and classical MM **(B),** the CIA ligand in complex with 1XOZ during the hybrid ML/MM **(C)** and classical MM **(D)** and the protein 1XOZ during the hybrid ML/MM **(E)** and classical MM **(F)** simulations for each replica. Each color represents a different replica. **(G)** Mean RMSD values over three replicas of 20 ns for the ligand CIA in solvent, the ligand CIA in complex and for the protein.

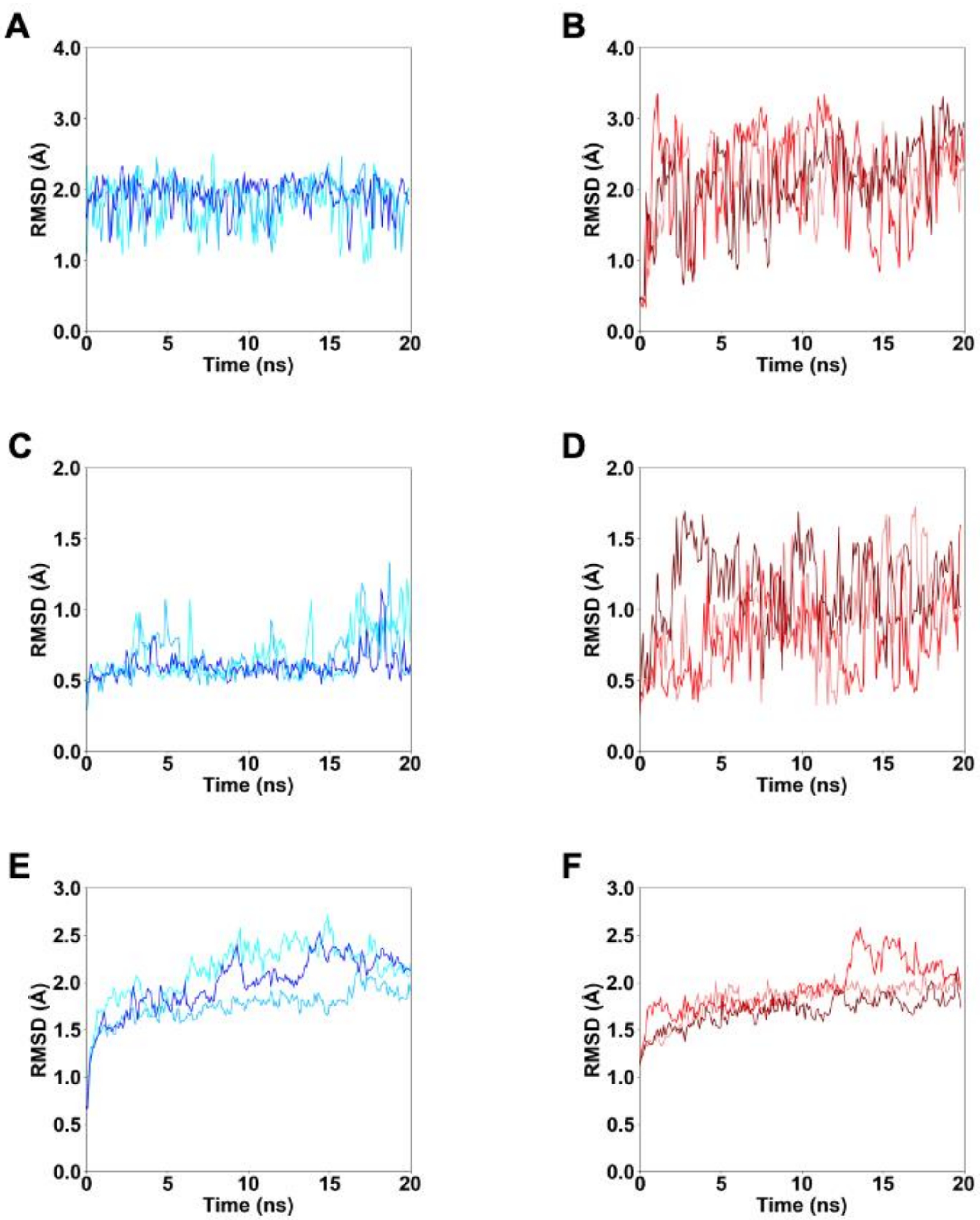

G

	System	ML/MM	MM
	AQ4 solvent	1.83 ± 0.02	1.99 ± 0.06
4HJO	AQ4 complex	0.64 ± 0.01	0.95 ± 0.02
	Protein	1.90 ± 0.02	1.82 ± 0.02

Figure S2. RMSD timeseries of the AQ4 ligand in solution during the hybrid ML/MM **(A)** and classical MM **(B),** the AQ4 ligand in complex with 4HJO during the hybrid ML/MM **(C)** and classical MM **(D)** and the protein 4HJO during the hybrid ML/MM **(E)** and classical MM **(F)** simulations for each replica. Each color represents a different replica. **(G)** Mean value of the RMSD over three replicas of 20 ns for the ligand AQ4 in solvent, the ligand AQ4 in complex and for the protein.

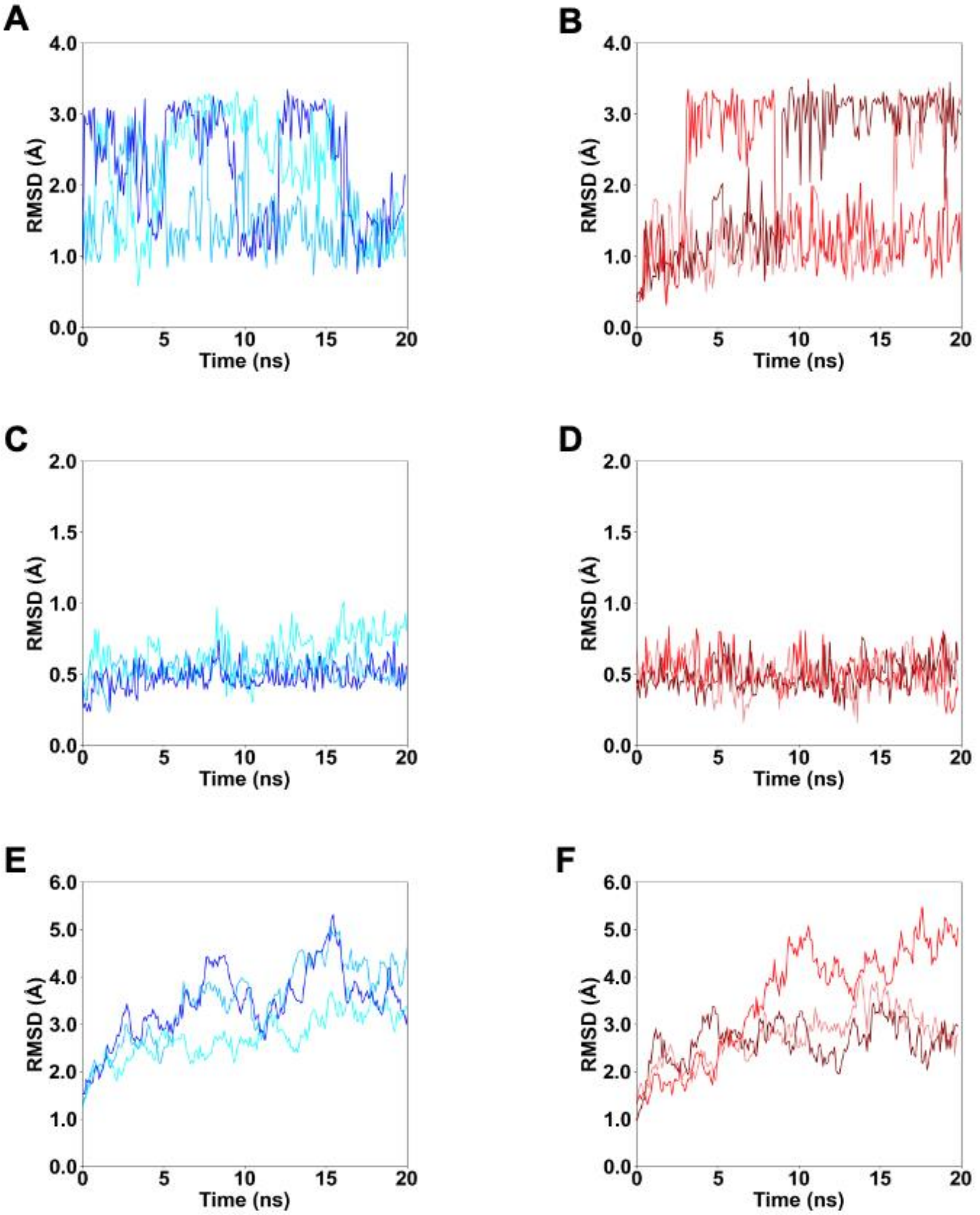

G

System		ML/MM	MM
6KPC	E3R solvent	1.94 ± 0.03	1.67 ± 0.06
	E3R complex	0.55 ± 0.01	0.49 ± 0.01
	Protein	1.99 ± 0.07	2.95 ± 0.06

Figure S3. RMSD timeseries of the E3R ligand in solution during the hybrid ML/MM **(A)** and classical MM **(B),** the E3R ligand in complex with 6KPC during the hybrid ML/MM **(C)** and classical MM **(D)** and the protein E3R during the hybrid ML/MM **(E)** and classical MM **(F)** simulations for each replica. Each color represents a different replica. **(G)** Mean value of the RMSD over three replicas of 20 ns for the ligand E3R in solvent, the ligand E3R in complex and for the protein.
